# Detecting Driver Drowsiness Based on Sensors: A Review

**DOI:** 10.3390/s121216937

**Published:** 2012-12-07

**Authors:** Arun Sahayadhas, Kenneth Sundaraj, Murugappan Murugappan

**Affiliations:** AI-Rehab Research Group, Universiti Malaysia Perlis (UniMAP), Kampus Pauh Putra, 02600 Arau, Perlis, Malaysia; E-Mails: kenneth@unimap.edu.my (K.S.); murugappan@unimap.edu.my (M.M.)

**Keywords:** driver drowsiness detection, transportation safety, hybrid measures, driver fatigue, artificial intelligence techniques, sensor fusion

## Abstract

In recent years, driver drowsiness has been one of the major causes of road accidents and can lead to severe physical injuries, deaths and significant economic losses. Statistics indicate the need of a reliable driver drowsiness detection system which could alert the driver before a mishap happens. Researchers have attempted to determine driver drowsiness using the following measures: (1) vehicle-based measures; (2) behavioral measures and (3) physiological measures. A detailed review on these measures will provide insight on the present systems, issues associated with them and the enhancements that need to be done to make a robust system. In this paper, we review these three measures as to the sensors used and discuss the advantages and limitations of each. The various ways through which drowsiness has been experimentally manipulated is also discussed. We conclude that by designing a hybrid drowsiness detection system that combines non-intusive physiological measures with other measures one would accurately determine the drowsiness level of a driver. A number of road accidents might then be avoided if an alert is sent to a driver that is deemed drowsy.

## 1. Introduction

According to available statistical data, over 1.3 million people die each year on the road and 20 to 50 million people suffer non-fatal injuries due to road accidents [1]. Based on police reports, the US National Highway Traffic Safety Administration (NHTSA) conservatively estimated that a total of 100,000 vehicle crashes each year are the direct result of driver drowsiness. These crashes resulted in approximately 1,550 deaths, 71,000 injuries and $12.5 billion in monetary losses [2]. In the year 2009, the US National Sleep Foundation (NSF) reported that 54% of adult drivers have driven a vehicle while feeling drowsy and 28% of them actually fell asleep [3]. The German Road Safety Council (DVR) claims that one in four highway traffic fatalities are a result of momentary driver drowsiness [4]. These statistics suggest that driver drowsiness is one of the main causes of road accidents.

A driver who falls asleep at the wheel loses control of the vehicle, an action which often results in a crash with either another vehicle or stationary objects. In order to prevent these devastating accidents, the state of drowsiness of the driver should be monitored. The following measures have been used widely for monitoring drowsiness:

1. Vehicle-based measures—A number of metrics, including deviations from lane position, movement of the steering wheel, pressure on the acceleration pedal, *etc.*, are constantly monitored and any change in these that crosses a specified threshold indicates a significantly increased probability that the driver is drowsy [5,6].
2. Behavioral measures—The behavior of the driver, including yawning, eye closure, eye blinking, head pose, *etc.*, is monitored through a camera and the driver is alerted if any of these drowsiness symptoms are detected [7–9].
3. Physiological measures—The correlation between physiological signals (electrocardiogram (ECG), electromyogram (EMG), electrooculogram (EoG) and electroencephalogram (EEG)) and driver drowsiness has been studied by many researchers [10–14].

Other than these three, researchers have also used subjective measures where drivers are asked to rate their level of drowsiness either verbally or through a questionnaire. The intensity of drowsiness is determined based on the rating [15,16].

These methods have been studied in detail and the advantages and disadvantages of each have been discussed. However, in order to develop an efficient drowsiness detection system, the strengths of the various measures should be combined into a hybrid system.

The organization of this paper is as follows: Section 2 discusses driver drowsiness in detail. Section 3 describes the simulated environment for drowsiness manipulation and Section 4 analyses the various methods of drowsiness manipulation for study purposes. Section 5 describes the different methods that have been studied for detecting driver drowsiness, Section 6 discusses on driving conditions and hybrid measures, and Section 7 concludes by presenting the benefit of fusing various measures to develop an efficient system.

## 2. Defining Drowsiness

The term “drowsy” is synonymous with sleepy, which simply means an inclination to fall asleep. The stages of sleep can be categorized as awake, non-rapid eye movement sleep (NREM), and rapid eye movement sleep (REM). The second stage, NREM, can be subdivided into the following three stages [17]:

Stage I: transition from awake to asleep (drowsy)
Stage II: light sleep
Stages III: deep sleep

In order to analyze driver drowsiness, researchers have mostly studied Stage I, which is the drowsiness phase. The crashes that occur due to driver drowsiness have a number of characteristics [18]:

- Occur late at night (0:00 am–7:00 am) or during mid-afternoon (2:00 pm–4:00 pm)
- Involve a single vehicle running off the road
- Occur on high-speed roadways
- Driver is often alone
- Driver is often a young male, 16 to 25 years old
- No skid marks or indication of braking

In relation to these characteristics, the Southwest England and the Midlands Police databases use the following criteria to identify accidents that are caused by drowsiness [5]:

- Blood alcohol level below the legal driving limit
- Vehicle ran off the road or onto the back of another vehicle
- No sign of brakes being applied
- Vehicle has no mechanical defect
- Good weather conditions and clear visibility
- Elimination of “speeding” or “driving too close to the vehicle in front” as potential causes
- The police officer at the scene suspects sleepiness as the primary cause

Statistics derived using these criteria cannot account fully for accidents caused by drowsiness because of the complexity involved; therefore, accidents that can be attributed to driver drowsiness may be more devastating than the statistics reveal. Hence, in order to avoid these types of accidents, it is necessary to derive effective measures to detect driver drowsiness and alert the driver.

## 3. Simulated Environment for Drowsiness Manipulation

**Figure 1.**
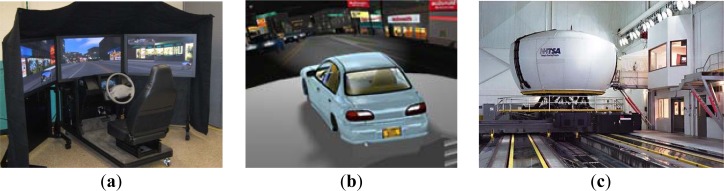
(**a**) MiniSim; (**b**) NADS 2; (**c**) NADS 1. Reproduced with permission from (Mini Sim: http://news-releases.uiowa.edu/2010/april/041210mini-sim.html; NADS 1: http://www.popsci.com/cars/gallery/2009-07/nads-1-worlds-most-advanced-driving-sim; NADS 2: http://www.nads-sc.uiowa.edu/sim_nads2.php).

It is not safe and ethical to make a drowsy driver drive on road. Hence, researchers have used simulated environments to carry out their experiments. Experimental control, efficiency, low cost, safety, and ease of data collection are the main advantages of using simulators [19,20]. The driving simulators can be broadly classified as: (1) Low-level simulators (Figure 1(a)) consisting of a computer, a monitor, a realistic cockpit, a steering wheel, manual gear box and pedals (clutch, brake and accelerator); (2) Mid-level (Fixed-base) simulators (Figure 1(b)) comprising of advanced imaging techniques, a large projection screen, a realistic car, and possibly a simple motion base and (3) High-level (Motion-based) simulators (Figure 1(c)) typically providing a view close to 360° and an extensive moving base [21].

One important limitation of using driving simulators is that the drivers do not perceive any risk. The awareness of being immersed in a simulated environment might give a behavior which is different than that on real road [22]. However, researchers have validated that driving simulators can create driving environment that are relatively similar to road experiments [23–25]. Researchers have observed behavioral [20,23], vehicle based [6] and physiological [24] similarity between simulated and on road experiments.

## 4. Drowsiness Manipulation for Study Purposes

One of the challenges in developing an efficient drowsiness detection system is how to obtain proper drowsiness data. Due to safety reasons, drowsiness cannot be manipulated in a real environment; thus, the drowsiness detection system has to be developed and tested in a laboratory setting. However, in a laboratory setting, the most reliable and informative data that pertains to driver drowsiness relies only on the way in which the driver falls into the drowsy state.

Driver drowsiness mainly depends on: (i) the quality of the last sleep; (ii) the circadian rhythm (time of day) and (iii) the increase in the duration of the driving task [11,26,27]. In some research experiments, the subjects were fully deprived of sleep, whereas they were only partially deprived of sleep in others [28]. In addition, some researchers recruited night shift workers as their subjects; in these cases, the subjects were totally deprived of sleep because the experiments were conducted in the morning [26,28]. Kokonozi *et al.* conducted an experiment in which they monitored the participants for 24 h before the experiment began to ensure that they were completely sleep-deprived [11]. In certain experiments, researchers partially deprived the subjects of sleep by allowing them to sleep for less than 6 h [14]. Peters *et al.* studied the same subjects during four consecutive days and considered the effects of no sleep deprivation, partial sleep deprivation and total sleep deprivation on their drowsiness level. They observed that, even in the case of partial sleep deprivation, the subjects tend to get drowsy after some time. Hence, the quality of the last sleep is an important criteria that influences drowsiness [29].

The performance of the driver deteriorates when physiological activity diminishes [30]. A circadian rhythm is used to refer to any biological variations or rhythms that occur in a cycle of approximately 24 h. These rhythms are self-sustaining (*i.e.*, free running) and will persist even when the organism is placed in an environment devoid of time cues, such as constant light or constant darkness [27]. Recent statistics from countries such as the United Kingdom, the United States, Israel, Finland, and France indicate that an increased number of vehicle accidents caused by driver drowsiness occurred during the peak drowsiness periods of 2:00 am to 6:00 am and 2:00 pm to 4:00 pm. During these time frames, the circadian rhythm shows higher chance of getting drowsy and drivers are three times more likely to fall asleep at these times than at 10:00 am or at 7:00 pm [31]. Liu *et al.* pointed out that the circadian rhythm produces small, but significant, changes in vehicle-based measures [5]. Researchers have asked subjects to drive between 2:30 pm and 5:30 pm in order to monitor drowsiness by measuring eyelid movement, ECG and EEG [14].

The duration of the driving task also plays a major role in influencing drowsiness. Otamani *et al.* found that sleep deprivation alone does not directly influence the brain signals that control drowsiness, whereas the duration of task has a strong influence [32]. Researchers have also inferred that prolonged driving on a monotonous environment stimulates drowsiness. In fact, it has been observed that the subjects can become drowsy within 20 to 25 min of driving [33]. This last finding, reported by Philip *et al.*, contradicts the observation made by Thiffault *et al.* that, in a real environment, the duration of the drive does not impact the performance during the first two hours [15]. In addition, researchers have found that drowsiness-related crashes are more probable in a monotonous environment than in a stimulating environment.

Therefore, there is a very high probability that a partially sleep-deprived driver will become drowsy when driving in a monotonous environment at a constant speed for three hours during a time when their circadian rhythm is low. This should be taken into consideration when designing an experiment relating to recording driver drowsiness.

## 5. Methods for Measuring Drowsiness

Researchers have used various methods to measure driver drowsiness. This section provides a review of the four most widely-used methods, among which the first method is measured either verbally or through questionnaire and the remaining three by means of various sensors.

### 5.1. Subjective Measures

Subjective measures that evaluate the level of drowsiness are based on the driver’s personal estimation and many tools have been used to translate this rating to a measure of driver drowsiness. The most commonly used drowsiness scale is the Karolinska Sleepiness Scale (KSS), a nine-point scale that has verbal anchors for each step, as shown in Table 1[32]. Hu *et al.* measured the KSS ratings of drivers every 5 min and used it as a reference to the EoG signal collected [28]. Portouli *et al.* evaluated EEG data by confirming driver drowsiness through both a questionnaire and a licensed medical practitioner [34]. Some researchers compared the self-determined KSS, which was recorded every 2 min during the driving task, with the variation of lane position (VLP) and found that these measures were not in agreement [35]. Ingre *et al.* determined a relationship between the eye blink duration and the KSS collected every 5 min during the driving task [26].

Researchers have determined that major lane departures, high eye blink duration and drowsiness-related physiological signals are prevalent for KSS ratings between 5 and 9 [26]. However, the subjective rating does not fully coincide with vehicle-based, physiological and behavioral measures.

Because the level of drowsiness is measured approximately every 5 min, sudden variations cannot be detected using subjective measures. Another limitation to using subjective ratings is that the self-introspection alerts the driver, thereby reducing their drowsiness level. In addition, it is difficult to obtain drowsiness feedback from a driver in a real driving situation. Therefore, while subjective ratings are useful in determining drowsiness in a simulated environment, the remaining measures may be better suited for the detection of drowsiness in a real environment.

### 5.2. Vehicle-Based Measures

Another method to measure driver drowsiness involves vehicle-based measurements. In most cases, these measurements are determined in a simulated environment by placing sensors on various vehicle components, including the steering wheel and the acceleration pedal; the signals sent by the sensors are then analyzed to determine the level of drowsiness. Liu *et al.*[5] published a review on current vehicle-based measures. Some researchers found that sleep deprivation can result in a larger variability in the driving speed [36]. However, the two most commonly used vehicle-based measures are the steering wheel movement and the standard deviation of lane position.

**Steering Wheel Movement (SWM)** is measured using steering angle sensor and it is a widely used vehicle-based measure for detecting the level of driver drowsiness [32,33,36]. Using an angle sensor mounted on the steering column, the driver’s steering behavior is measured. When drowsy, the number of micro-corrections on the steering wheel reduces compared to normal driving [37]. Fairclough and Graham found that sleep deprived drivers made fewer steering wheel reversals than normal drivers [36]. To eliminate the effect of lane changes, the researchers considered only small steering wheel movements (between 0.5° and 5°), which are needed to adjust the lateral position within the lane [32]. Hence, based on small SWMs, it is possible to determine the drowsiness state of the driver and thus provide an alert if needed. In a simulated environment, light side winds that pushed the car to the right side of the road were added along a curved road in order to create variations in the lateral position and force the drivers to make corrective SWMs [33]. Car companies, such as Nissan and Renault, have adopted SWMs but it works in very limited situations [38]. This is because they can function reliably only at particular environments and are too dependent on the geometric characteristics of the road and to a lesser extent on the kinetic characteristics of the vehicle [38].

**Standard Deviation of Lane Position (SDLP)** is another measure through which the level of driver drowsiness can be evaluated [26]. In a simulated environment, the software itself gives the SDLP and in case of field experiments the position of lane is tracked using an external camera. Ingre *et al.* conducted an experiment to derive numerical statistics based on SDLP and found that, as KSS ratings increased, SDLP (meters) also increased [26]. For example, KSS ratings of 1, 5, 8, and 9 corresponded to SDLP measurements of 0.19, 0.26, 0.36 and 0.47, respectively. The SDLP was calculated based on the average of 20 participants; however, with some drivers, the SDLP did not exceeded 0.25 m even for a KSS rating of 9. In the above experiment by performing correlation analysis on a subject to subject basis significant difference is noted. Another limitation of SDLP is that it is purely dependent on external factors like road marking, climatic and lighting conditions. In summary, many studies have determined that vehicle-based measures are a poor predictor of performance error risk due to drowsiness. Moreover, vehicular-based metrics are not specific to drowsiness. SDLP can also be caused by any type of impaired driving, including driving under the influence of alcohol or other drugs, especially depressants [39–41].

### 5.3. Behavioral Measures

A drowsy person displays a number of characteristic facial movements, including rapid and constant blinking, nodding or swinging their head, and frequent yawning [7]. Computerized, non-intrusive, behavioral approaches are widely used for determining the drowsiness level of drivers by measuring their abnormal behaviors [42]. Most of the published studies on using behavioral approaches to determine drowsiness, focus on blinking [43–45]. PERCLOS (which is the percentage of eyelid closure over the pupil over time, reflecting slow eyelid closures, or “droops”, rather than blinks) has been analyzed in many studies [8,46–48]. This measurement has been found to be a reliable measure to predict drowsiness [46] and has been used in commercial products such as Seeing Machines [49] and Lexus [50]. Some researchers used multiple facial actions, including inner brow rise, outer brow rise, lip stretch, jaw drop and eye blink, to detect drowsiness [9,42]. However, research on using other behavioral measures, such as yawning [51] and head or eye position orientation [52,53], to determine the level of drowsiness is ongoing (Table 2).

The main limitation of using a vision-based approach is lighting. Normal cameras do not perform well at night [43]. In order to overcome this limitation, some researchers have used active illumination utilizing an infrared Light Emitting Diode (LED) [43]. However, although these work fairly well at night, LEDs are considered less robust during the day [54]. In addition, most of the methods have been tested on data obtained from drivers mimicking drowsy behavior rather than on real video data in which the driver gets naturally drowsy. Mostly, image is acquired using simple CCD or web camera during day [55] and IR camera during night [56] at around 30 fps. After capturing the video, some techniques, including Connected Component Analysis, Cascade of Classifiers or Hough Transform, Gabor Filter, Haar Algorithm are applied to detect the face, eye or mouth [8,42,44,56]. After localizing the specific region of interest within the image, features such as PERCLOS, yawning frequency and head angle, are extracted using an efficient feature extraction technique, such as Wavelet Decomposition, Gabor Wavelets, Discrete Wavelet Transform or Condensation Algorithm [7,42,44,56]. The behavior is then analyzed and classified as either normal, slightly drowsy, highly drowsy through the use of classification methods such as support vector machine, fuzzy classifier, neural classifier and linear discriminant analysis [7,42–44]. However, it has been found that the rate of detecting the correct feature, or the percentage of success among a number of detection attempts, varies depending on the application and number of classes. The determination of drowsiness using PERCLOS and Eye Blink has a success rate of close to 100% [43] and 98% [45], respectively. However it has to be noted that, the high positive detection rate achieved by [43] was when the subjects didn’t wear glasses. Likewise, as most researchers conducted their experiments in simulated environment they achieved a higher success rate. The positive detection rate decreased significantly when the experiment was carried out in a real environment [15].

Another limitation of behavioral measure was brought out in an experiment conducted by Golz *et al.* They evaluated various drowsiness monitoring commercial products, and observed that driver state cannot be correlated to driving performance and vehicle status based on behavioral measures alone [57].

### 5.4. Physiological Measures

As drivers become drowsy, their head begins to sway and the vehicle may wander away from the center of the lane. The previously described vehicle-based and vision based measures become apparent only after the driver starts to sleep, which is often too late to prevent an accident.

However, physiological signals start to change in earlier stages of drowsiness. Hence, physiological signals are more suitable to detect drowsiness with few false positives; making it possible to alert a drowsy driver in a timely manner and thereby prevent many road accidents.

Many researchers have considered the following physiological signals to detect drowsiness: electrocardiogram (ECG), electromyogram (EMG), electroencephalogram (EEG) and electro-oculogram (EoG) (Table 3). Some researchers have used the EoG signal to identify driver drowsiness through eye movements [12,28,61]. The electric potential difference between the cornea and the retina generates an electrical field that reflects the orientation of the eyes; this electrical field is the measured EoG signal. Researchers have investigated horizontal eye movement by placing a disposable Ag-Cl electrode on the outer corner of each eye and a third electrode at the center of the forehead for reference [28]. The electrodes were placed as specified so that the parameters - Rapid eye movements (REM) and Slow Eye Movements (SEM) which occur when a subject is awake and drowsy respectively, can be detected easily [30].

The heart rate (HR) also varies significantly between the different stages of drowsiness, such as alertness and fatigue [13,63]. Therefore, heart rate, which can be easily determined by the ECG signal, can also be used to detect drowsiness. Others have measured drowsiness using Heart Rate Variability (HRV), in which the low (LF) and high (HF) frequencies fall in the range of 0.04–0.15 Hz and 0.14–0.4 Hz, respectively [12,58]. HRV is a measure of the beat-to-beat (R-R Intervals) changes in the heart rate. The ratio of LF to HF in the ECG decreases progressively as the driver progresses from an awake to a drowsy state [14,58].

The Electroencephalogram (EEG) is the physiological signal most commonly used to measure drowsiness. The EEG signal has various frequency bands, including the delta band (0.5–4 Hz), which corresponds to sleep activity, the theta band (4–8 Hz), which is related to drowsiness, the alpha band (8–13 Hz), which represents relaxation and creativity, and the beta band (13–25 Hz), which corresponds to alertness [10,59,60,62]. A decrease in the power changes in the alpha frequency band and an increase in the theta frequency band indicates drowsiness. Akin *et al.* observed that the success rate of using a combination of EEG and EMG signals to detect drowsiness is higher than using either signal alone [10].

The measurement of raw physiological signals is always prone to noise and artifacts due to the movement that is involved with driving. Hence, in order to eliminate noise, various preprocessing techniques, such as low pass filter, digital differentiators, have been used (Table 2). In general, an effective digital filtering technique would remove the unwanted artifacts in an optimal manner [64]. A number of statistical features are then extracted from the processed signal using various feature extraction techniques, including Discrete Wavelet Transform (DWT) and Fast Fourier Transform (FFT) [10,59,60]. The extracted features are then classified using Artificial Neural Networks (ANN), Support Vector Machines (SVM), Linear Discriminant Analysis (LDA), or other similar methods [12,28,61].

The reliability and accuracy of driver drowsiness detection by using physiological signals is very high compared to other methods. However, the intrusive nature of measuring physiological signals remains an issue to be addressed. To overcome this, researchers have used wireless devices to measure physiological signals in a less intrusive manner by placing the electrodes on the body and obtaining signals using wireless technologies like Zigbee [65], Bluetooth [66]. Some researchers have gone further ahead by measuring physiological signals in a non intrusive way; by placing electrodes on the steering wheel [67,68] or on the driver’s seat [67,69]. The signals obtained were then processed in android based smart phone devices [70,71] and the driver was alerted on time. The accuracy of a non-intrusive system is relatively less due to movement artifacts and errors that occur due to improper electrode contact. However, researchers are considering to use this because of its user friendliness. In recent years, experiments are conducted to validate non-intrusive systems [68,69]. The advantages and disadvantages of the different type of measures are summarized in Table 4.

## 6. Discussion

The various measures of driver drowsiness reviewed in this work are based purely on the level of drowsiness induced in the subject, which, in turn, depends on the time of day, duration of the task and the time that has elapsed since the last sleep. However, when developing a better drowsiness detection system, several other issues need to be addressed; the two most important ones are discussed below.

### 6.1. Comparison of Simulated and Real Driving Conditions

It is not advisable to force a drowsy driver to drive on roads. Consequently, many experiments have been conducted in simulated environments and the results of the experiments are then elaborately studied. Dinges *et al.* presented various challenges involved in real time drowsiness detection [46]. The subjective self-assessment of drowsiness can only be obtained from subjects in simulated environments. In real conditions, it is unfeasible to obtain this information without significantly distracting the driver from their primary task. Some researchers have conducted experiments to confirm the validity of simulated driving environments. For example, Blana *et al.* observed that the mean lateral displacement of the vehicle from the center of the roadway, obtained in real and simulated environments is statistically different for speeds higher than 70 km/h. This finding implies that real-road drivers feel less safe at higher speeds and, as a result, increase their lateral distance. The drivers in a simulated environment, however, did not appear to perceive this risk [72]. Most experiments using behavioral measures are conducted in a simulated environment and the results indicate that it is a reliable method to detect drowsiness. However, in real driving conditions, the results might be significantly different because a moving vehicle can present challenges such as variations in lighting, change in background and vibration noise, not to mention the use of sunglasses, caps, *etc.* Philip *et al.* compared drowsiness in simulated and real conditions and concluded that it can be equally studied in both environments but the reaction time and the sleepiness self-evaluation are more affected in a simulated environment which provides a more monotonous task [15]. Engstorm *et al.* observed that the physiological workload and steering activity was higher in a real environment. This result can be interpreted as an indication of increased effort, which seems reasonable given the higher actual risk in real traffic [73]. Hence, while developing a drowsiness detection system, the simulated environment should be as close to a replica of the real environment as possible.

### 6.2. Hybrid Measures

**Figure 2.**
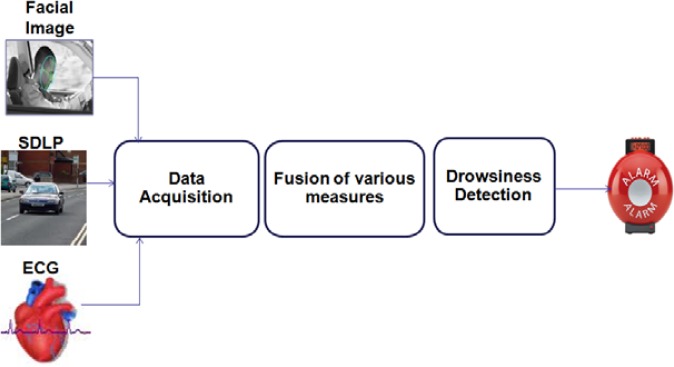
A sample hybrid drowsiness detection system using multiple sensors.

Each method used for detecting drowsiness has its own advantages and limitations. Vehicle-based measures are useful in measuring drowsiness when a lack of vigilance affects vehicle control or deviation. However, in some cases, there was no impact on vehicle-based parameters when the driver was drowsy [26], which makes a vehicle-based drowsiness detection system unreliable. Behavioral measures are an efficient way to detect drowsiness and some real-time products have been developed [74]. However, when evaluating the available real-time detection systems, Lawrence *et al.* observed that different illumination conditions affect the reliability and accuracy of the measurements [74]. Physiological measures are reliable and accurate because they provide the true internal state of the driver; however, their intrusive nature has to be resolved. Among all physiological parameters investigated, ECG can be measured in a less intrusive manner. EEG signals require a number of electrodes to be placed on the scalp and the electrodes used for measuring EoG signals are placed near the eye which can hinder driving. Non-obtrusive physiological sensors to estimate the drowsiness of drivers are expected to become feasible in the near future [70,75]. The advantages of physiological measures and the increasing availability of non-intrusive measurement equipment make it beneficial to combine physiological signals with behavioral and vehicle-based measures. A sample drowsiness detection system developed by combining ECG signals, standard deviation of lane position and facial images is shown in Figure 2.

Few research studies are attempting to detect driver drowsiness by the fusion of different methods [14,76–78]. Cheng *et al.* combined behavioral measures and vehicle based measures and concluded that the reliability and accuracy of the hybrid method was significantly higher than those using single sensors [78]. Guosheng *et al.* used a mixture of subjective, behavioral (PERCLOS) and physiological measures (ECG, EEG) to detect drowsiness and found that this combination resulted in a significantly higher success rate than any individual metric. The average square error while removing physiological features were 1.2629, while the average square error for fusion was 0.5269 [14].

Although hybrid systems using different sensors have not been tested in a real environment, it would be interesting to investigate the ability to detect drowsiness using a combination of physiological signals with other measurements.

## 7. Conclusions

In this paper, we have reviewed the various methods available to determine the drowsiness state of a driver. Although there is no universally accepted definition for drowsiness, the various definitions and the reasons behind them were discussed. This paper also discusses the various ways in which drowsiness can be manipulated in a simulated environment. The various measures used to detect drowsiness include subjective, vehicle-based, physiological and behavioral measures; these were also discussed in detail and the advantages and disadvantages of each measure were described. Although the accuracy rate of using physiological measures to detect drowsiness is high, these are highly intrusive. However, this intrusive nature can be resolved by using contactless electrode placement. Hence, it would be worth fusing physiological measures, such as ECG, with behavioral and vehicle-based measures in the development of an efficient drowsiness detection system. In addition, it is important to consider the driving environment to obtain optimal results.

## Figures and Tables

**Table 1.** Karolinska sleepiness scale (KSS).

**Rating**	**Verbal descriptions**
1	Extremely alert
2	Very alert
3	Alert
4	Fairly alert
5	Neither alert nor sleepy
6	Some signs of sleepiness
7	Sleepy, but no effort to keep alert
8	Sleepy, some effort to keep alert
9	Very sleepy, great effort to keep alert, fighting sleep

**Table 2.** List of previous works on driver drowsiness detection using behavioral measures.

**Ref.**	**Sensor used**	**Drowsiness Measure**	**Detection techniques**	**Feature Extraction**	**Classification**	**Positive Detection rate**
[55]	CCD micro camera with Infra-Red Illuminator	Pupil	Ada-boost	Red eye effect, Texture detection method	Ratio of eye-height and eye-width	92%
[43]	Camera and Infra-Red Illuminator	PERCLOS, eye closure duration, blink frequency, and 3 other	Two Kalman filters for pupil detection	Modification of the algebraic distance algorithm for conics Approximation & Finite State Machine	Fuzzy Classifier	Close to 100%
[7]	CCD camera	Yawning	Gravity-center template and grey projection	Gabor wavelets	LDA	91.97%
[42]	Digital Video camera	Facial action	Gabor filter	Wavelet Decomposition	SVM	96%
[44]	Fire wire camera and webcam	Eye Closure Duration & Freq of eye closure	Hough Transform	Discrete Wavelet Transform	Neural Classifier	95%
[9]	Camera	Multi Scale dynamic features	Gabor filter	Local Binary Pattern	Ada boost	98.33%
[56]	IR Camera	Eye State	Gabor filter	Condensation algorithm	SVM	93%
[45]	Simple Camera	Eye blink	Cascaded Classifiers Algorithm detects face and Diamond searching lgorithm to trace the face	Duration of eyelid closure, No. of continuous blinks, Frequency of eye blink	Region Mark Algorithm	98%
[8]	Camera with IR Illuminator	PERCLOS	Haar Algorithm to detect face	Unscented Kalman filter algorithm	SVM	99%

**Table 3.** List of previous works on driver drowsiness detection using physiological signals.

**Ref.**	**Sensors**	**Preprocessing**	**Feature Extraction**	**Classification**	**Classification accuracy (%)**
[12]	EEG, ECG, EoG	Optimal Wavelet Packet, Fuzzy Wavelet Packet	The Fuzzy MI-based Wavelet-Packet Algorithm	LDA, LIBLINEAR, KNN, SVM	95–97% (31 drivers)
[58]	ECG	Band Pass Filter	Fast Fourier Transform (FFT)	Neural Network	90% (12 drivers)
[59]	EEG	Independent Component Analysis Decomposition	Fast Fourier Transform	Self-organizing Neural Fuzzy Inference Network	96.7% (6 drivers)
[10]	EEG, EMG	Band Pass Filter & Visual Inspection	Discrete Wavelet Transform (DWT)	Artificial Neural Network (ANN) Back Propogation Algorithm (Awake, Drowsy, Sleep)	98–99% (30 subjects)
[60]	EEG	Low pass filter 32 Hz	512 point Fast Fourier Transform with 448 point overlap	Mahalanobis distance	88.7% (10 subjects)
[28]	EoG, EMG	Filtering & Thresholding	Neighborhood search	SVM	90% (37 subjects)
[61]	EEG, EoG, EMG	Low pass pre Filter and Visual Inspection	Discrete Wavelet Transform	ANN	97–98% (10 subjects)
[62]	EEG	Least mean square algorithm and Visual Inspection	Wavelet packet analysis with Daubechies 10 as mother wavelet	Hidden Markov Model	84% (50 subjects)

**Table 4.** Advantages and limitations of various measures.

**Refs.**	**Measures**	**Parameters**	**Advantages**	**Limitations**
[26,35]	Subjective measures	Questionnaire	Subjective	Not possible in real time
[5,72]	Vehicle based measures	Deviation from the lane position Loss of control over the steering wheel movements	Nonintrusive	Unreliable
[15,54]	Behavioral Measures	Yawning Eye closure Eye blink Head pose	Non-intrusive; Ease of use	Lighting condition Background
[67,69]	Physiological measures	Statistical & energy features derived from ECG EoG EEG	Reliable; Accurate	Intrusive
