# Correction: Sahayadhas, A., et al. Detecting Driver Drowsiness Based on Sensors: A Review. *Sensors* 2012, *12*, 16937–16953

**DOI:** 10.3390/s21020451

**Published:** 2021-01-11

**Authors:** Arun Sahayadhas, Kenneth Sundaraj, Murugappan Murugappan

**Affiliations:** AI-Rehab Research Group, Universiti Malaysia Perlis (UniMAP), Kampus Pauh Putra, Arau 02600, Perlis, Malaysia; kenneth@unimap.edu.my (K.S.); murugappan@unimap.edu.my (M.M.)

The authors wish to make the following corrections to this paper [1]:

The sentences of paragraph 4 in Section 4, “Researchers have also inferred that prolonged driving on a monotonous environment stimulates drowsiness. In fact, it has been observed that the subjects can become drowsy within 20 to 25 min of driving [33]. This last finding, reported by Philip et al., contradicts the observation made by Thiffault et al. that, in a real environment, the duration of the drive does not impact the performance during the first two hours [15].”, should be updated to, “Researchers have also inferred that prolonged driving in a monotonous environment facilitates drowsiness. In fact, it has been observed that subjects can become hypovigilant and even drowsy within 20 to 25 min of driving in simulated monotonous conditions [33]. Other authors have reported that, in a real environment, the duration of the drive alone does not impact the performance of driving [15].”

The sentences in the original version did not convey the intended message. Hence, minor changes have been made by reframing the sentences. The authors would like to apologize for any inconvenience caused to the readers by these changes.
